# Discovering disease-causing pathogens in resource-scarce Southeast Asia using a global metagenomic pathogen monitoring system

**DOI:** 10.1073/pnas.2115285119

**Published:** 2022-03-01

**Authors:** Jennifer A. Bohl, Sreyngim Lay, Sophana Chea, Vida Ahyong, Daniel M. Parker, Shannon Gallagher, Jonathan Fintzi, Somnang Man, Aiyana Ponce, Sokunthea Sreng, Dara Kong, Fabiano Oliveira, Katrina Kalantar, Michelle Tan, Liz Fahsbender, Jonathan Sheu, Norma Neff, Angela M. Detweiler, Christina Yek, Sokna Ly, Rathanak Sath, Chea Huch, Hok Kry, Rithea Leang, Rekol Huy, Chanthap Lon, Cristina M. Tato, Joseph L. DeRisi, Jessica E. Manning

**Affiliations:** ^a^Laboratory of Malaria and Vector Research, National Institute of Allergy and Infectious Diseases, National Institutes of Health, Bethesda, MD 20892;; ^b^International Center of Excellence in Research, National Institute of Allergy and Infectious Diseases, National Institutes of Health, 12101 Phnom Penh, Cambodia;; ^c^National Center for Parasitology, Entomology, and Malaria Control, Ministry of Health, 12101 Phnom Penh, Cambodia;; ^d^Chan Zuckerberg Biohub, San Francisco, CA 94158;; ^e^Department of Population Health and Disease Prevention, University of California, Irvine, CA 92697;; ^f^Biostatistics Research Branch, National Institute of Allergy and Infectious Diseases, National Institutes of Health, Bethesda, MD 20892;; ^g^Chan Zuckerberg Initiative, Redwood City, CA 94063;; ^h^Kampong Speu District Referral Hospital, 05251 Chbar Mon, Cambodia;; ^i^Department of Biochemistry and Biophysics, University of California, San Francisco, CA 94143

**Keywords:** metagenomics, Southeast Asia, vector-borne disease, next-generation sequencing, pathogen surveillance

## Abstract

Understanding the regional pathogen landscape and surveillance of emerging pathogens is key to mitigating epidemics. Challenges lie in resource-scarce settings, where outbreaks are likely to emerge, but where laboratory diagnostics and bioinformatics capacity are limited. Using metagenomic next-generation sequencing (mNGS), we identified a variety of vector-borne, zoonotic, and emerging pathogens responsible for undifferentiated fevers in a periurban population in Cambodia. From March 2019 to October 2020, we enrolled 464 febrile patients (and 23 afebrile persons) aged 6 mo to 65 y presenting to a large periurban hospital in Cambodia. We collected sera and prepared sequencing libraries from extracted pathogen RNA for unbiased metagenomic sequencing and subsequent bioinformatic analysis on the global cloud-based platform, CZID (“IDseq”). We employed multivariable regression models to evaluate pathogen risk factors associated with undifferentiated febrile illness. mNGS identified vector-borne pathogens as the largest clinical category with dengue virus (124 of 489) as the most abundant pathogen. Underappreciated zoonotic pathogens, such as *Plasmodium knowlesi*, leptospirosis, and coinfecting HIV were also detected. Early detection of chikungunya virus presaged a larger national outbreak of more than 6,000 cases. Pathogen-agnostic mNGS investigation of febrile persons in resource-scarce Southeast Asia is feasible and revealing of a diverse pathogen landscape. Coordinated and ongoing mNGS pathogen surveillance can better identify the breadth of endemic, zoonotic, or emerging pathogens and deployment of rapid public health response.

## Significance

Metagenomic pathogen sequencing offers an unbiased approach to characterizing febrile illness. In resource-scarce settings with high biodiversity, it is critical to identify disease-causing pathogens in order to understand burden and to prioritize efforts for control. Here, metagenomic next-generation sequencing (mNGS) characterization of the pathogen landscape in Cambodia revealed diverse vector-borne and zoonotic pathogens irrespective of age and gender as risk factors. Identification of key pathogens led to changes in national program surveillance. This study is a “real world” example of the use of mNGS surveillance of febrile individuals, executed in-country, to identify outbreaks of vector-borne, zoonotic, and other emerging pathogens in a resource-scarce setting.

A global pathogen surveillance network can best identify emerging and underlying pathogens if it employs pathogen-agnostic detection methods, such as metagenomic next-generation sequencing (mNGS), and is decentralized to include low-resource settings that are often biodiversity hotspots at increased risk for disease outbreaks (1–3). Lack of diagnostics in these areas makes undifferentiated febrile illnesses difficult to diagnose and treat, much less confirm and report for global public health awareness. In Southeast Asia, where a quarter of the world’s population resides, rapid but heterogeneous economic development juxtaposes low-resource and high-resource areas, causing high cross-border mobility of persons for economic opportunities. In Cambodia and Laos, laboratory testing for nonmalarial fevers is limited, particularly in rural and periurban areas where simple diagnostics like dengue rapid tests may not be available (4). In many instances, healthcare providers make diagnoses and empiric treatment decisions based on symptoms, so the responsible pathogen is rarely identified.

Syndromic diagnosis is an epidemiological pitfall in Southeast Asia because the true scope of pathogen diversity remains poorly defined. From limited decade-old surveillance data of febrile Cambodians, *Plasmodium* infections made up more than 50% of the responsible pathogens followed by pathogenic *Leptospira* (9.4%), influenza virus (8.9%), and dengue virus (DENV) (6.3%) (5). In a separate serosurvey, one-third of febrile Cambodian patients had antibodies to rickettsiae that cause scrub typhus (via chiggers containing *Orientia tsutsugamushi*), endemic typhus (via rat fleas *Xenopsylla cheopia* carrying *Rickettsia typhi*), spotted fever (via ticks carrying *Rickettsia rickettsii*), and murine typhus (via cat fleas *Ctenocephalides felis* carrying *Rickettsia felis*) (6, 7). Entomological studies of field-collected ticks, mosquitos, and fleas in Cambodia have revealed high biodiversity of potential disease-carrying vectors, including underappreciated *Bartonella* spp. (8, 9). Other serosurveys of bats, domestic pigs, and birds in Cambodia demonstrated the presence of antibodies to other zoonotic viruses, including Nipah virus, hepatitis E, Japanese encephalitis virus, and West Nile virus with potential for spillover into the human population (10–12).

In these settings of high pathogen diversity, monitoring with pathogen-agnostic tools, such as mNGS, is ideal but typically not available in-country to provide results within an actionable time frame. Examples of mNGS identifying pathogens in patients are limited to clinical research programs in developed countries (13–15). However, it is clear that broadly applied and timely mNGS in any population can lead to a better understanding of the overall pathogen landscape, which has direct implications for disease containment methods in the event of an outbreak (16, 17). Here, as an initial step in a low-resource setting in Asia, we describe implementation of mNGS surveillance using an open-source cloud-based bioinformatics tool to identify pathogens in sera from febrile individuals in periurban Cambodia.

## Results

### Clinical Characteristics of Febrile Participants in Cambodia.

**Fig. 1.**
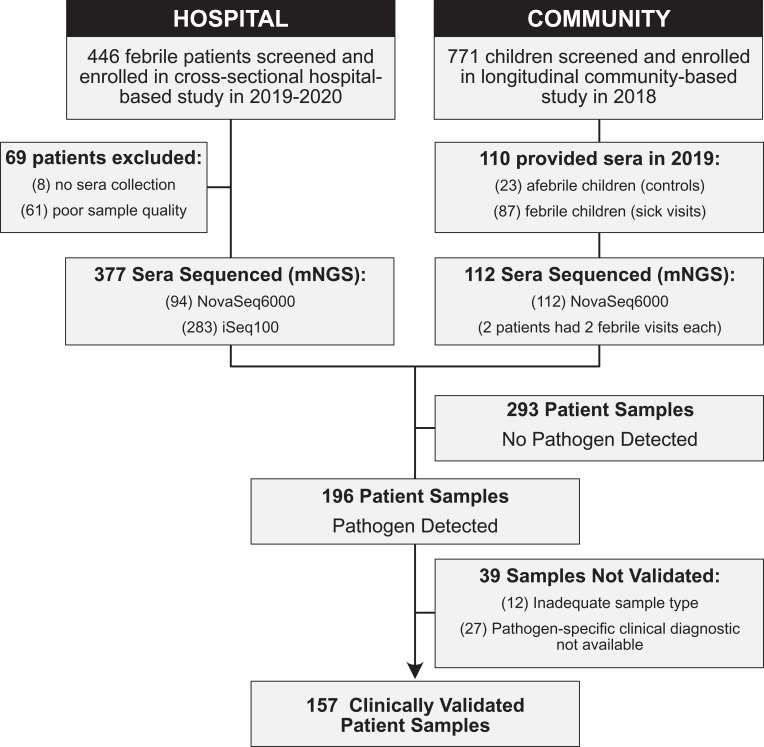
Study flowchart. Flow of enrolled febrile patients through two clinical studies defined as hospital (cross-sectional febrile patient hospital-based cohort) and community (longitudinal pediatric community-based cohort).

From March 2019 to October 2020, a total of 464 patients presenting with fever were screened, enrolled, and contributed sera for mNGS (377 patients in hospital-based cohort and 87 in community-based cohort) plus sera from 23 afebrile controls presenting for “healthy” follow-up used to establish a background for bioinformatics analyses (Fig. 1). Demographic and clinical characteristics are detailed in Table 1; notably, the participants are young with the median age in the hospital cohort at 10 y (interquartile range, IQR 12), and 6 y (IQR 4) in the community cohort. The predominant symptom reported in both studies was headache 52.4% (256 of 487). Of the adults, 67.7% (61 of 90) were employed in nonagricultural settings while the remainder were farmers or unemployed. In only the hospital cohort, approximately half of participants reported insect exposure, primarily mosquitos (211 of 376). Nearly three-quarters of participants reported animal exposure (275 of 376). The most common animal exposures included dogs, cats, and chickens, with some rare reports of exposure to pigs and horses.

**Table 1.** Baseline demographic and clinical characteristics

Characteristic	Hospital	Community	Total
*n*	377	110	487
Male	207 (55)	56 (51)	263 (54)
Age, y (median, IQR)	10, 12	6, 4	8, 10
Year of fever			
2019	196 (52)	110 (100)	306 (63)
Attends school	146 (39)	64 (58)	210 (43)
Attends work	75 (20)	0 (0)	75 (15)
Socioeconomic status			
Very poor	16 (4)	0, 0.0	16 (3)
Lower	178 (47)	22 (20)	200 (41)
Middle	181 (48)	88 (80)	269 (55)
Upper	1 (0.3)	0 (0)	1 (0.2)
Risk factors			
Coil use	22 (60)	70 (64)	295 (61)
Insecticide use	191 (51)	60 (54.5)	251 (52)
Larvicide use	28 (7)	27 (24.5)	55 (11)
Insecticide-treated bed net use	313 (83)	99 (90)	412 (85)
Self-reported animal contact	275 (73)	N/A	275 (73)
Self-reported insect contact^*^	211 (56)	N/A	211 (56)
Symptoms^†^			
Aching	131 (35)	N/A	131 (35)
Chills	167 (44)	N/A	167 (44)
Cough	175 (46),	N/A	175 (46)
Headache	236, (63)	20 (18)	256 (52)
Joint pain	N/A	1 (1)	1 (1)
Mouth sores	88 (23)	N/A	88 (23)
Muscle pain	N/A	4 (4)	4 (1)
Runny nose	66 (17.5)	N/A	66 (18)
Heart palpitations	120 (32)	N/A	120 (32)
Rash	81 (21.5)	0, 0.0	81 (17)
Clinical laboratory data^‡^			
*n*	240	47	287
White blood cell count			
Low (<6 10^9^/L)	90 (37.5)	19 (40.4)	109 (38)
Normal (6–16 10^9^/L)	137 (57.1)	27 (57.4)	164 (57)
High (>16 10^9^/L)	13 (5.4)	1 (2.1)	14 (5)
Lymphocyte			
Low (<3.5 10^9^/L)	199 (83)	43 (91.5)	242 (84)
Normal (3.5–11 10^9^/L)	39 (16)	4(8.5)	43 (15)
High (>11 10^9^/L)	2 (1)	0 (0)	2 (1)
Neutrophil			
Low (< 1 10^9^/L)	12 (5)	2 (4)	14 (5)
Normal (1–7 10^9^/L)	167 (70)	35 (75)	200 (70)
High (>7 10^9^/L)	61 (25)	10 (21)	73 (25)
Platelets			
Low (<200 10^9^/L)	106 (44.2)	13 (28)	119 (41.5)
Medium (200–550 10^9^/L)	133 (55.4)	32 (72)	167 (58)
High (>550 10^9^/L)	1 (0.4)	0 (0)	1 (0.3)

These data are in *n*, % unless otherwise stated

* This question was specifically asked in the hospital study questionnaire but not in the community study questionnaire and the only insects reported were mosquito and spider.

^†^Twenty-three control patients from the Community Study were afebrile and did not have symptoms.

^‡^Not all patients had complete blood counts because study physician decided based on clinical necessity.

### mNGS Characterization of the Pathogen Landscape in Febrile Cambodians.

**Fig. 2.**
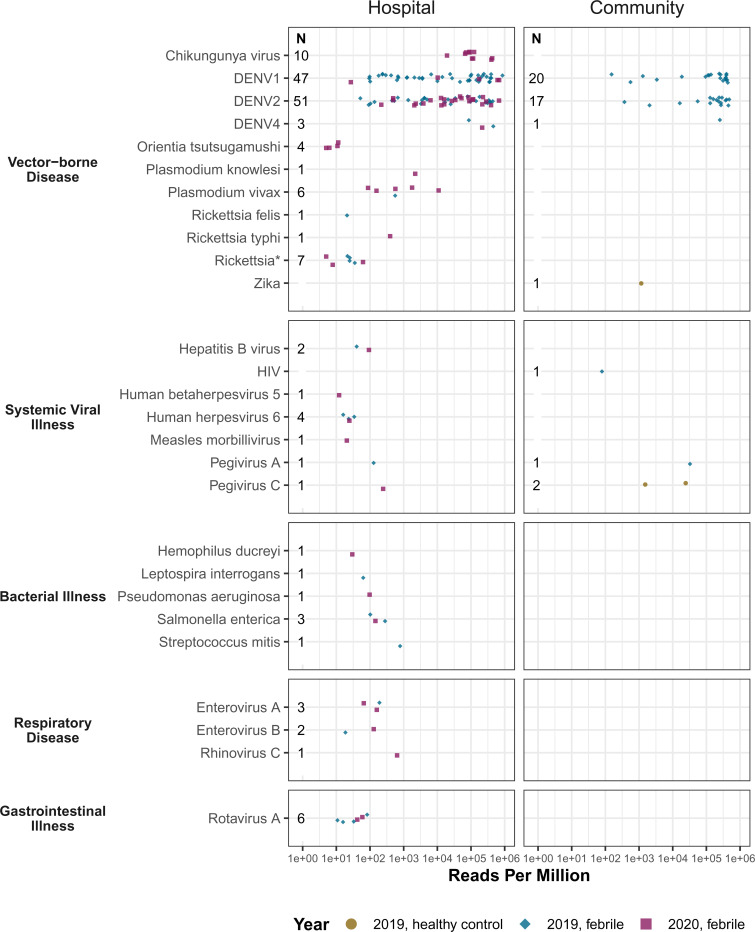
Microbial landscape identified from serum samples of febrile Cambodian participants. Identified pathogens in sera by clinical category, rpM, and study setting. Each circle represents a pathogen in 2019 and each diamond a pathogen in 2020. Pathogens found in afebrile control participants are denoted by a square. An asterisk (*) denotes genus-level confidence only.

The composite of identified pathogens in both cohorts is shown in Fig. 2. From 489 sera samples, 203 pathogens were detected, yielding a pathogen result rate of 41.5%. Seven participants were coinfected with multiple pathogens (7 of 489; 1.4%). Vector-borne disease was the most prevalent clinical category for mNGS analysis of sera from febrile patients. This clinical category included DENV (138 of 489), most abundant, followed by rickettsiae (13 of 489), chikungunya virus (CHIKV) (10 of 489), and *Plasmodium vivax* (6 of 489). The second highest clinical category was systemic viral illness notably including hepatitis and pegiviruses (8 of 489).

### Pathogen Serosurveillance Findings in Clinical and Regional Contexts.

Here, we describe select pathogens in greater detail pertinent to clinical and genomic epidemiology of the Southeast Asian region.

#### Dengue virus.

Dengue was responsible for the greatest disease burden in our study (138 DENV^+^ cases of 489 febrile cases) due to the largest DENV outbreak documented in Cambodian history in 2019. The predominant DENV serotype of the outbreak in Kampong Speu province was DENV1 (*SI Appendix*, Table S2). Seventy-one percent (48 of 67) of DENV1 sequences identified, aligned to DENV1, accession no. MF033254.1 from 2016 DENV1 outbreak in Singapore. Phylodynamic analyses will be presented elsewhere.

#### Rickettsia.

While rickettsial diseases are easily treated with oral doxycycline, the challenge is timely diagnosis and access to serological and molecular testing for confirmation. In Laos, a country of similar climate and socioeconomic status as Cambodia, 7% (122 of 1,871) of febrile patients were positive for scrub typhus, 1% murine typhus (10 of 1,849), and 1% undetermined *Rickettsia* spp. combined with *R. felis* (9 of 1,849) (18). Here, in our study, four patients were positive for *O. tsutsugamushi*, highly homologous to accession no. CP044031.1 from Zhejiang province, China and to accession no. LS398552.1 from Udon Thani, Thailand (19). Our mNGS pipeline identified one case of *R. felis*, one of *R. typhi* and seven cases of the genus *Rickettsia* without clinical confirmation of species-level data.

#### Chikungunya virus.

In July 2020, we identified 10 cases of CHIKV in Kampong Speu Province where patients presented with symptoms of fever, rash, shaking chills, and arthralgias. mNGS analysis revealed CHIKV as the clinical etiology after initial diagnoses of DENV were made based on patients’ presenting symptoms. These sequences aligned closely with three urban Asian lineage sequences from Thailand (accession nos. MN075149.1, MN630017.1, and MK468801.1). CHIKV PCR was then added to national surveillance and it was noted that the outbreak spread rapidly to 21 other provinces in Cambodia, affecting at least 6,000 people by the end of September 2020 despite implementation of vector control (20).

#### Zika virus.

Zika virus (ZIKV) circulates at low levels in Thailand and Vietnam; however, almost no active cases have been reported in Laos and Cambodia even during the global epidemic in 2015 to 2016 (21, 22). Since 2010, only one prospective case of active ZIKV infection was detected in Cambodia, notably Kampong Speu province (23). In the present study, sera from an otherwise asymptomatic 8-y-old female was positive for ZIKV with 20.1× coverage depth and 98.8% coverage breadth closely aligned with to accession no. MF996804.1, a Thai case of microcephaly, with 99.2% sequence similarity. These information indicate that ZIKV in Cambodia has regional sequence similarities to Thailand, possibly related to high cross-border traffic between the two countries despite little ZIKV detected in Cambodia (24). Another possibility is a separate enzootic ZIKV transmission cycle maintained in nonhuman primates given recent evidence of ZIKV in stump-tailed macaques in Thailand (25).

#### Plasmodium *spp.*

Cambodia is in the preelimination stage for all malarial species with a specific goal to eliminate *Plasmodium falciparum* by 2025 (26). mNGS identified six cases of very low parasitemia (down to 16 parasites per miroliter) with *P. vivax*, initially missed on microscopy or rapid test. *P. vivax* has replaced *P. falciparum* as the most prevalent form of malaria in Southeast Asia, particularly in Cambodia where eradicative liver-stage treatment of *P. vivax* with primaquine has not yet been widely adopted (4). mNGS also identified *Plasmodium knowlesi* in a forest worker, previously diagnosed with *Plasmodium malariae* using blood-smear microscopy. This pathogen identification led to retrospective mNGS assessment of other *P. malariae* cases and the addition of *P. knowlesi* PCR to national surveillance. Given human encroachment and deforestation in Southeast Asia, there is ample opportunity for spread of zoonotic malaria, such as *P. knowlesi* typically found in nonhuman primates, that may endanger elimination goals (4).

#### Leptospira *interrogans.*

Leptospirosis is an underappreciated health threat in Southeast Asia. In nearby Kampong Cham province, 2.5% (17 of 630) of all fevers in 27 rural to semirural villages were confirmed as acute leptospirosis infection via IgM serology and microagglutination testing (27). In November 2019, a 7-y-old female with a fever of 38.5 °C presented with a headache and abdominal pain. mNGS identified *Leptospira interrogans* at 62.9 reads per million (rpM), with 99.7% homology to CP048830.1. Due to limited in-country diagnostic testing, no further testing was performed, but clinical examination confirmed the presence of conjunctival effusion, a specific feature of leptospirosis.

#### HIV-1 and DENV coinfection.

An 8-y-old female of Vietnamese descent presented to the hospital with a 39 °C fever and mNGS analysis revealed a possible coinfection of DENV2 and HIV. The low sequence coverage (14%) of a Vietnamese HIV genome, accession no. FJ185253.1, was likely due to the sequencing space used on the high number of DENV2 reads (DENV2: 368 rpM, 99% sequence coverage breadth and 17.1× depth versus HIV: 78.1 rpM, 14% coverage and 1× depth) for this sample (28). However, remapping all reads belonging to the *Lentivirus* genus resulted in a more comprehensive assessment with 33% coverage of the Vietnamese HIV-1 viral genome (accession no. FJ185246. 1) at a depth of 3.47×, with greatest homology to a Thai HIV-1 strain, accession no. LC114832.1, from a female sex worker. The mNGS results were confirmed by clinically validated HIV 1/2 antibody tests, and the patient subsequently initiated antiretroviral therapy.

### Risk Modeling of Contracting Vector-Borne Disease.

**Fig. 3.**
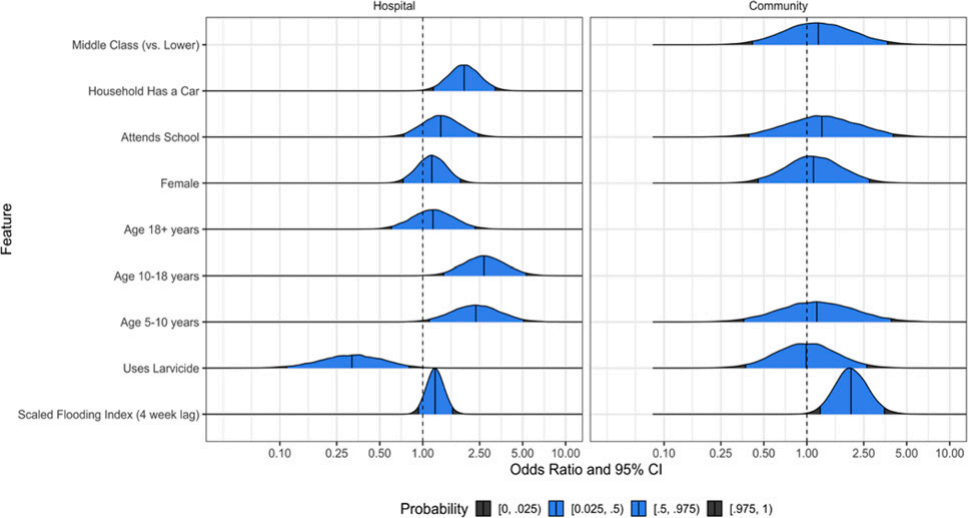
Odds ratio that a patient encounter was attributable to infection by a vector-borne pathogen. Results of multivariate analyses in both patient populations to identify risk factors of contracting vector-borne pathogens.

In adjusted analyses, the odds that a patient encounter in the hospital study was attributable to infection by a vector-borne pathogen was higher if the household owned a car (adjusted odds ratio [aOR] 1.95, 95% confidence interval [CI] 1.19 to 3.21) or if they were 5 to 18 y of age (for 5 to 10 y of age; aOR 2.35, 95% CI 1.11 to 5.06; aOR 2.68 for 10 to 18 y of age; 1.4 to 5.29) (Fig. 3 and *SI Appendix*, Table S3). Use of larvicide was negatively associated with the likelihood that a patient encounter was due to a vector-borne pathogen (aOR 0.32, 95% CI 0.11 to 0.80). In the community study, living near surface flooding, using a Scaled Flooding Index (4-wk lag), significantly increased the likelihood of vector-borne infection (aOR 2.04, 95% CI 1.24 to 3.49). Larvicide (e.g., temephos to which *Aedes* spp. is typically resistant in Cambodia) was not associated with the odds that a patient encounter was due to a vector-borne disease (aOR 0.99 95% CI 0.0.37 to 2.63).

**Fig. 4.**
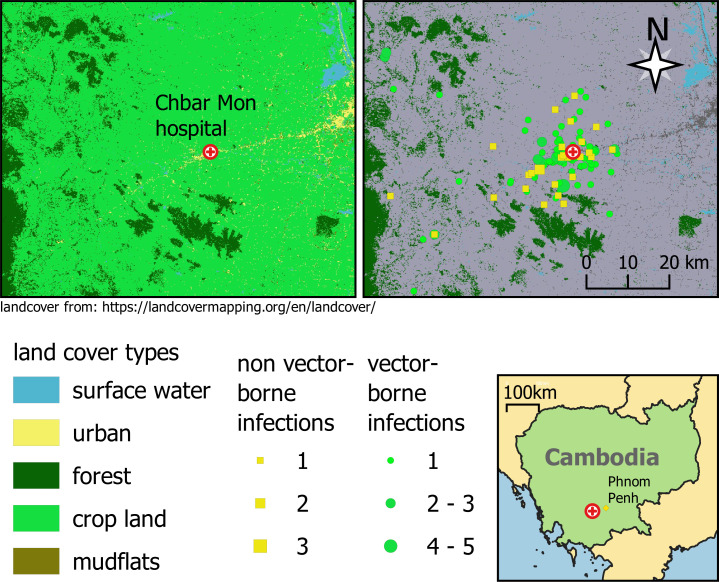
Study site and land use map. Patient locations classified by land use and vector-borne disease status.

Crop land was the predominant land-cover type for participants’ homes (89%; 426 to 476) (Fig. 4) with urban as the next most common (10%; 49 of 476). Urban participants were more likely to have nonvector borne diseases (13%; 4 of 30) than vector-borne pathogens (9%; 15 of 162); however, there were still participants from primarily urban areas with CHIK, DENV1, DENV2, or ZIKV infections (*SI Appendix*, Table S1). Interestingly, 92% (125 of 135) of DENV cases were from crop land (*SI Appendix*, Table S4). Formal analyses by each disease outcome were not pursued because of small overall counts. Exploratory univariate analysis of the environmental indices (EIs) indicated that only the surface flooding index was associated with any of the disease outcomes (*SI Appendix*, Fig. S2).

## Discussion

Metagenomic NGS serosurveillance from periurban Cambodia revealed a diverse pathogen landscape, rich in underappreciated vector-borne and zoonotic pathogens, responsible for febrile disease. In this prospective, cross-sectional mNGS study, we identified common and confounding pathogens and demonstrated the feasibility and usefulness of a decentralized metagenomic sequencing pipeline. Actionable mNGS surveillance is challenging in a resource-scarce settings, but genomics-informed pathogen epidemiology—that is, otherwise lacking in Cambodia and other similar settings—is important and globally relevant given major demographic and socioeconomic shifts underway in the region that may increase the likelihood for disease epidemics (4, 29). Our study provides a granular analysis of changing pathogen dynamics than prior surveillance with predetermined targeted diagnostics like PCR (5, 30, 31). The hierarchy of species abundance identified here is likely attributed to current malaria elimination campaigns, heterogenous socioeconomic development, increased dengue transmission, and ongoing human migration (4, 32).

Over the past two decades, the importance of vector-borne pathogens as drivers of epidemics and as emerging pathogens cannot be discounted despite current threats posed by novel respiratory pathogens. The detection of primarily vector-borne pathogens in this study is relevant as genomic surveillance becomes the foundation of global health security. The Asian strain of ZIKV evolved to enhance infectivity of humans and mosquitos via a single alanine-to-valine substitution that increased NS1 antigenemia, ultimately resulting in epidemics as early as 2007 in Micronesia and later in the Americas linked to microcephaly (33). Prior CHIK outbreaks were traced to a single mutation in 2005 allowing increased fitness of CHIK in *Aedes albopictus* mosquitos, and thus conferring epidemic potential of the virus in humans (34). Today, autochthonous CHIK transmission and outbreaks occur in increasingly warmer temperate zones like Europe (35). mNGS also recently identified novel vector-borne pathogens, including the tick-borne flaviviruses like Alongshan and highly fatal mosquito-borne orthobunyaviruses like Cristoli, Umbre, and others (36–38). These emerging pathogens were identified in high-resource areas where clinical staff had access to mNGS technology. Logistical and bureaucratic delays in shipping samples out of a country may translate to the establishment and spread of a pathogen in the interim.

To that end, timely contribution of pathogen genomic information from resource-limited settings is critical to the future success of pathogen identification based on genomic sequence data in an increasingly connected world, exemplified by GISAID and GENBANK during the SARS-CoV-2 pandemic (3). The lack of publicly available sequence data of clinically relevant pathogens, such as DENV and CHIK in Southeast Asia, is stark given the regional magnitude of infections by these pathogens.

Our mNGS surveillance primarily identified vector-borne pathogens; therefore, our risk models aimed to inform deductive algorithms for undifferentiated fevers in the region. Judicious use of mNGS surveillance would not entail sequencing every undifferentiated fever that is presumed to be dengue. With dengue being the most common diagnosis attributed to fevers in pediatric patients, we aimed to include demographic, behavior, and ecological data that might stratify risk of a vector-borne disease pathogen versus other pathogens in the hospital-based cohort of all ages. Exposure to animals and occupations did not stratify to any risk, but younger age, household car ownership (a surrogate of socioeconomic status), and absence of larvicide use led to increased risk of vector-borne diseases. Advances in land-cover analysis now permit disease risk assessment of a population based on their environment. Here, living near surface flooding increased vector-borne disease risk, and surprisingly, DENV cases originated primarily in crop zones that often border urban zones, corroborating previous claims that DENV transmission in Southeast Asia is both rural and urban (39). Even with these tools to aid diagnostic algorithms, it is evident from our data that assigning microbial etiology to undifferentiated fever based on symptoms and demographic data are difficult given the presence of diverse pathogens, the shifting of socioeconomic patterns, and the ongoing transformation of land cover.

Limitations in the study included the sampling strategy of sera or whole blood alone, primarily for operational purposes in the early establishment of this pathogen mNGS detection pipeline. To that point, exclusive use of sera contributed to our pathogen detection rate of 40%, likely overestimating vector-borne pathogens to the detriment of respiratory and gastrointestinal pathogens that may be poorly detected in the blood. Other clinical studies illustrate the strengths of an mNGS approach in a variety of sample types and the ability of an unbiased approach to detect “unexpected” but clinically relevant pathogens (13, 40–42). Since completion of the data analysis presented here, our mNGS monitoring efforts now include nasopharyngeal swabs, in addition to ongoing blood sampling. To date, the addition of nasopharyngeal sampling to our mNGS surveillance study has led to timely recovery of entire SARS-CoV-2 genomes, with and without enrichment, for variant identification (43, 44). Fortunately, genome recovery of most viruses was straightforward from sera, but sampling limitations remain for other taxa; for example, the optimal sample type to identify and speciate *Rickettsiae* is buffy coat, as opposed to sera, because the bacteria are intracellular (45). Other challenges included identification of less-abundant bacterial pathogens, attributable to limited coverage offered by the iSeq, variable host contamination, different library preparation (e.g., DNA-based instead of RNA-based), and again, sample type. The cross-sectional study design limited our ability to see if a patient’s clinical course evolved over a longer period of time, and the lack of blood culture capabilities at this hospital did not allow comparison of mNGS to standard diagnostic techniques for bacterial pathogen identification. However, we strived for actionable data, from either a clinical or public health standpoint, and succeeded in cases of *Plasmodium* spp., HIV, CHIK, and other pathogens. The cost of sequencing is declining, while the efficiency of sequencing workflows is increasing, but mNGS analysis of pathogens is still more expensive than targeted diagnostics like PCR or culture (1).

Until now, the majority of sequencing and analysis of biological samples collected in Cambodia and other resource-limited settings was outsourced to the Global North. To overcome challenges in reagent procurement, internet connectivity, and lack of advanced bioinformatics training, we built a robust infrastructure to mitigate these issues while also relying upon a precurated, rapid bioinformatics pipeline to build in-country expertise that allowed the entire sample collection, processing, and mNGS analysis to happen in a public Cambodian laboratory.

As a result, our ongoing, in-country metagenomic sequencing pipeline and capacity-building provides continuous monitoring of common and emerging pathogens for actionable interventions when possible. While the world looks to bolster real-time, genomics-informed pathogen surveillance networks to monitor COVID-19 variants and other emerging pathogens, challenges remain to establish critical “nodes” in biodiverse, resource-scarce areas (3, 46). Yet, as shown here, mNGS pathogen surveillance in these settings is feasible, revealing of diverse microbial landscapes, and paramount to the future of global health security.

## Materials and Methods

### Ethics.

This study was approved by the Cambodian National Ethics Committee on Human Research and the NIH Institutional Research Board. Written informed consent was obtained from the participant or the parent or guardian of participants under 18 y of age enrolled in this study. Additionally, informed assent was obtained from individuals aged 14 to 17 y of age in addition to their parents’ consent. This study was registered at https://clinicaltrials.gov as NCT04034264 and NCT03534245.

### Enrollment.

Screening and enrollment of febrile patients occurred in both the inpatient and outpatient departments at Kampong Speu District Referral Hospital, a 120-bed periurban hospital in Chbar Mon, Cambodia ∼90- to 120-min driving distance from Phnom Penh, the capital city of Cambodia (*SI Appendix*, Table S1). Overall, participants were required to 1) be 6 mo to 65 y of ag, and 2) have a measured fever equal to or greater than 38 °C in previous 24 h (see https://clinicaltrials.gov for full criteria). Some participants were already enrolled in a longitudinal, community-based cohort of children, 2 to 9 y of age, under semiactive surveillance because study participants were told to notify study coordinator and present to the hospital when they have a fever, called a “sick visit,” that was considered nested cross-sectional time point within the longitudinal cohort and therefore are called “community” here. The remaining participants were enrolled in a cross-sectional hospital-based febrile cohort established in July 2019 (referred to as “hospital” and considered passive surveillance because patients first presented to the hospital with fever and were then asked to participate). We used sera from 23 healthy, afebrile children from the community cohort undergoing scheduled follow-up sampling to establish a baseline pathogen profile and use as background for bioinformatics analysis but not in the Bayesian modeling analysis. Demographics, clinical, and risk factor data were stored in a REDCAP database. Locational data were collected using Garmin GPS devices and Google Earth.

### Sample Collection and Nucleic Acid Extraction.

At enrollment, ∼5 mL of whole blood was collected (except 2 mL collected from those under 2 y old). Sera was isolated and stored in cryovials with an equal volume of 2× DNA/RNA Shield (Zymo Research) at −20 °C and transported from the Kampong Speu Hospital laboratory to the Cambodian National Center for Parasitology Entomology and Malaria Control in Phnom Penh, Cambodia. Pathogen RNA was isolated from sera using Quick-RNA MicroPrep Kit (Zymo Research) and DNase-treated.

### Library Preparation.

mNGS libraries were prepared from isolated pathogen RNA and converted to cDNA Illumina libraries using the NEBNext Ultra II RNA Library Prep Kit (New England BioLabs). Human rRNA was depleted via FastSelect -rRNA HMR (Qiagen). ERCC Spike-In Controls (ThermoFisher) were used to indicate potential library preparation errors and to calculate input RNA mass. The initial samples (*n* = 208) were sequenced on a NovaSeq6000 (Illumina) instrument as part of a pilot wet laboratory training at the Chan Zuckerberg BioHub in San Francisco, CA, and then the remainder of the study (*n* = 279) was performed on an iSeq100 (Illumina) in Phnom Penh, Cambodia, using 150-nucleotide paired-end sequencing. Water controls were included in each library preparation.

### Bioinformatic Analysis.

Raw fastq files were uploaded to the CZID (IDseq) portal, a cloud-based, open-source bioinformatics platform, to identify microbes from metagenomic data (http://czid.org/) (47). Potential pathogens were distinguished from commensal flora and contaminating microbial sequences from the environment by establishing a *z*-score metric based on a background distribution derived from 16 nontemplate control libraries. Data were normalized to unique rpM input reads for each microbe at both species and genus levels. Taxa with *z*-score less than 1, an average base pair alignment of less than 50 base pairs, an e-score less than 1e-10, and rpM less than 10 were removed from analysis.

### Clinical Validation.

Pathogens for which clinical testing capabilities were available in-country were validated to include RT-PCR of Hepatitis B, *Plasmodium* spp., DENV, CHIK, and ZIKV, serology of HIV 1/2 antibodies or blood-smear examination of *Plasmodium* infections by World Health Organization-certified microscopists. Validation testing for other pathogens is underway or being developed. Samples were considered to have “no pathogen hit” if they meet QA/QC standards but no resulting pathogenic organisms were identified with appropriate thresholds in place.

### Spatial and Environmental Data.

Land-cover data for Cambodia were downloaded from Open Development Cambodia (https://opendevelopmentcambodia.net). The data come from the Regional Land Cover Monitoring System at a resolution of 30 m by 30 m and were from 2016 (the most recent year we could find at this resolution). We used freely available satellite imagery (Google Earth) to ensure that the land cover data matched the reality on the ground. Participant village locations were then plotted on top of the land-cover map. To summarize and quantify land-cover types, we created 1-km buffers around the geographic coordinates for participant villages and extracted land cover characteristics for each participant using the Zonal Histogram function in QGIS (v3.16.5: https://qgis.org). We then categorized each participant according to the land-cover type that predominated around their village location, and tabulated land-cover types according to disease outcomes. EI for surface water and vegetation were extracted from Moderate Resolution Imaging Spectroradiometer (MODIS) products (MOD13Q1/MYD13Q1 250 m AQUA/TERRA 16-d composites). A normalized flooding index (NDFI), the normalized differential vegetation index (NDVI), and the enhanced vegetation index (EVI) were all extracted for this analysis (48, 49). NDFI gives an indication of surface water, NDVI gives an indication of surface vegetation, and EVI is an improvement on NDVI in that it is less sensitive to atmospheric conditions and forest canopies. The data were downloaded for each 16-d time interval (from July 2018 to May 2020) using a 1-km buffer around the home of each patient in the dataset. The visit date of each participant was then used to align the EI values for each participant. EI values from the 16-d period leading up to a participant visit were used for analyses.

### Statistical Analysis.

The primary endpoint is identification of pathogen sequences via IDseq analysis in serum samples from febrile individuals treated at the Kampong Speu District Referral Hospital. On average, we found 25 to 40% of the monthly febrile cases were attributable to vector-borne disease. As such, we decided to determine which demographic variables, risk factors, and climate data were associated with vector-borne pathogen identification using a Bayesian logistic regression model. For our feature coefficients, we used a weakly informative prior and a MCMC sampler to determine the posterior distribution of the coefficients. We plotted the marginal coefficient densities and display the posterior medians along with 95% CIs. We fit two separate models: one for the hospital cohort and one for the community cohort. Most, but not all, features are present in both models. More details about variable selection, model diagnostics, and model sensitivity may be found in the supplemental material (*SI Appendix*). Of note, healthy or afebrile controls, for whom sera was sequenced for bioinformatics background purposes, were not included in the model despite some having detectable pathogens as they were not febrile with demonstrable clinical symptoms.

## Supplementary Material

Supplementary File

## Data Availability

All genome sequence data from this study have been submitted to the National Center for Biotechnology Information Sequence Read Archive (Bioproject ID PRJNA681566). All bioinformatics code is available on https://github.com (under https://github.com/chanzuckerberg/idseq-workflows) and all wet laboratory bench protocols are updated at https://docs.google.com/document/d/1RtNQc1D4or_ys7OxCCBjh4SDIdy7JaI4IE7if8EkHgE/edit. Further information on how to use 1/2 reaction volumes and FastSelect are available upon request. All other study data are included in the main text and *SI Appendix*.
