# Supplementary material for: Discovering disease-causing pathogens in resource-scarce Southeast Asia using a global metagenomic pathogen monitoring system
Source: Proc Natl Acad Sci U S A. 2022 Mar 1;119(11):e2115285119. doi: 10.1073/pnas.2115285119 (PMC8931249; doi:10.1073/pnas.2115285119)
Supplement: Supplementary File [file pnas.2115285119.sapp.pdf]

Supplementary Information for

Discovering disease-causing pathogens in resource-scarce Southeast Asia using a global metagenomic pathogen monitoring system

Jennifer A. Bohl[1,2], Sreyngim Lay[2,3], Sophana Chea[2,3], Vida Ahyong[4], Daniel M. Parker[5], Shannon Gallagher[6], Jonathan Fintzi[6], Somnang Man[2,3], Aiyana Ponce[1], Sokunthea Sreng[2,3], Dara Kong[2,3], Fabiano Oliveira[1], Katrina Kalantar[8], Michelle Tan[4], Liz Fahsbender[8], Jonathan Sheu[8], Norma Neff[4], Angela M. Detweiler[4], Sokna Ly[2,3], Rathanak Sath[2,9], Chea Huch[3], Hok Kry[9], Rithea Leang[3], Rekol Huy[3], Chanthap Lon[1,2], Cristina M. Tato[4], Joseph L. DeRisi*[4,10], Jessica E. Manning[1,2]

1 - Laboratory of Malaria and Vector Research, National Institute of Allergy and Infectious Diseases, National Institutes of Health, Bethesda, Maryland, USA

2 – International Center of Excellence in Research, National Institute of Allergy and Infectious Diseases, National Institutes of Health, Phnom Penh, Cambodia

3 – National Center for Parasitology, Entomology, and Malaria Control, Ministry of Health, Phnom Penh, Cambodia

4 – Chan Zuckerberg Biohub, San Francisco, California, USA

5 – University of California, Irvine, California USA

6– Biostatistics Research Branch, National Institute of Allergy and Infectious Diseases, National Institutes of Health, Bethesda, Maryland, USA

8 – Chan Zuckerberg Initiative, Redwood City, California, USA

9 – Kampong Speu District Referral Hospital, Chbar Mon, Cambodia

10 – University of California, San Francisco, California USA

Paste Joseph L. DeRisi
Email: joe@derisilab.ucsf.edu

This PDF file includes:

Figures S1
Tables S1 to S2
Metagenomics Methods Supplement Text
Statistical Supplement Text
SI References

Supplementary Information Text

Supplemental Table 1. Admissions, Referrals and Deaths at Kampong Speu District Referral Hospital, 2019 – 2020

	2019				2020			
Cases	< 5yrs	5-10 yrs	10-18 yrs	> 18 yrs	< 5yrs	5-10 yrs	10-18 yrs	> 18 yrs
OPD	2696	3263	4362	18490	2438	3320	6732	24502
IPD	1523	1265	2758	7805	1439	600	2426	8531
Refer cases	19	68	113	495	105	201	289	1050
Death	9	0	11	126	14	5	10	106

Supplemental Table 2. Cambodian dengue serotypes detected from March 2019 to December 2020 and closet regional homologous sequence.

Season	Serotypes Detected	Accession number (number of samples)	Location	Date	Co-infection
March to May 2019 (Dry Season)	DENV1	MF033260.1 (1)	Singapore	2016	N/A
		MF033254.1 (4)	Singapore	2016	HIV
	DENV2	LC410185.1 (2)	Thailand	2016	N/A
June to November 2019 (Wet Season)	DENV1	MF033254.1 (39)	Singapore	2016	N/A
		MF033260.1 (2)	Singapore	2016	N/A
		MK905537.1 (1)	Henan province, China	2019	N/A
		MN923084.1 (1)	China	2019	Rickettsia felis

	DENV2	FJ639718.1 (1)	Cambodia	2008	N/A
		KF955398.1 (1)	Cambodia	2008	N/A
		KU517847.1 (5)	Philippines	2015	N/A
		KU666944.1 (3)	Malaysia	2014	N/A
		KX452016.1 (5)	Malaysia	2014	N/A
		KY921905.1 (7)	Singapore	2015	N/A
		LC410185.1 (7)	Thailand	2016	N/A
		MH110564.1 (3)	Hangzhou, China	2017	N/A
		MH827546.1 (3)	China	2017	N/A
		MN328061.1 (1)	Bangladesh	2019	Hepatitis B virus
		MN923109.1 (2)	Chinese traveler from Cambodia	2019	N/A
	DENV4	KP792537.2 (1)	Singapore	2011	N/A
		KY924607.1 (1)	Vietnam	2016	N/A
		MK614090.1 (1)	Guangzhou, China	2018	N/A
December to May 2020 (Dry Season)	DENV1	MN444623.1 (3)	Zhejiang province, China	2019	N/A
		MN923092.1 (1)	China	2019	N/A
		MF033254.1 (1)	Singapore	2016	Measles morbillivirus

	DENV2	MN923109.1 (2)	Chinese traveler from Cambodia	2019	N/A
June to December 2020 (Wet Season)	DENV1	MN444623.1 (1)	Zhejiang province, China	2019	N/A
		LC428054.1 (1)	Vietnam	2017	N/A
		MF033254.1 (1)	Singapore	2013	N/A
	DENV2	KX452016.1 (1)	Malaysia	2014	N/A
		KX452018.1 (1)	Malaysia	2014	N/A
		MH827546.1 (13)	China	2017	N/A
		MK564480.1 (1)	Guangzhou, China	2016	N/A
		MN923109.1 (3)	Chinese traveler from Cambodia	2019	N/A
		MN923115.1 (2)	Chinese traveler from Cambodia	2019	N/A
	DENV4	MK614090.1 (2)	Guangzhou, China	2018	N/A

Supplemental Table 2.

Table 2. Adjusted odds ratios for risk factors of a patient encounter being attributable to vector born disease.

Risk Factors	Adjusted odds ratio	95% CI
Scaled flooding index		
Hospital	1.22	0.94 - 1.61
Community	2.04	1.24-3.49
Uses larvicide		
Hospital	0.32	0.11-0.8
Community	0.99	0.37-2.63
Age 5–10 years		
Hospital	2.35	1.11-5.06
Community	1.17	0.36-3.9
Age 10–18 years		
Hospital	2.68	1.4-5.29
Community	N/A	N/A
Age 18+ years		
Hospital	1.18	0.6-2.32
Community	N/A	N/A
Female		
Hospital	1.16	0.73-1.83
Community	1.11	0.46-2.74
Attends school		
Hospital	1.34	0.74-2.44
Community	1.27	0.39-4.02
Household has a car		
Hospital	1.95	1.19-3.21
Community	N/A	N/A
Middle class (vs. lower)		
Hospital	N/A	N/A
Community	1.2	0.42-3.66

Supplemental Table 3. Dominant land cover types around home villages of study participants (11 patients did not have reliable GPS point).

		Crop	Forest	Urban	Count
vector-borne disease	Chikungunya virus	6	0	4	10
	DENV1	60	0	5	65
	DENV2	58	0	5	63
	DENV2	3	0	0	3

	DENV4	4	0	0	4
	Orientia tsutsugamushi	4	0	0	4
	Plasmodium vivax	6	0	0	6
	Rickettsia spp.	5	0	0	5
	Rickettsia typhi	1	0	0	1
	Zika	0	0	1	1
	Vector-borne disease total	147	0	15	162
		0			
non-vector borne disease	Enterovirus A	3	0	0	3
	Enterovirus B	2	0	0	2
	Hemophilus ducreyi	1	0	0	1
	Hepatitis B virus	1	0	0	1
	HIV	0	0	1	1
	Human betaherpesvirus 5	1	0	0	1
	Human herpesvirus 6	4	0	0	4
	Leptospira interrogans	1	0	0	1
	Pegivirus A	1	0	1	2
	Pegivirus C	1	0	2	3
	Pseudomonas aeruginosa	1	0	0	1
	Rhinovirus C	1	0	0	1
	Rotavirus A	5	0	0	5
	Salmonella enterica	3	0	0	3
	Streptococcus mitis	1	0	0	1
	non-vector-borne disease total	26	0	4	30
		0			
no pathogen		253	1	30	284
	combined total	426	1	49	476

Figure S1. Temporal Trends in Pathogens in Community and Hospital Cohorts

A) Hospital Cohort

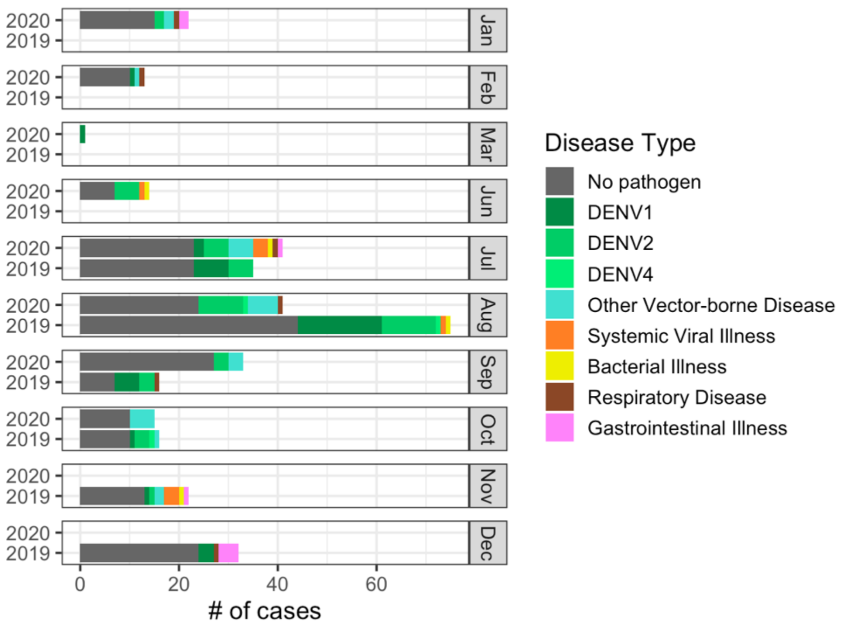

B) Community Cohort

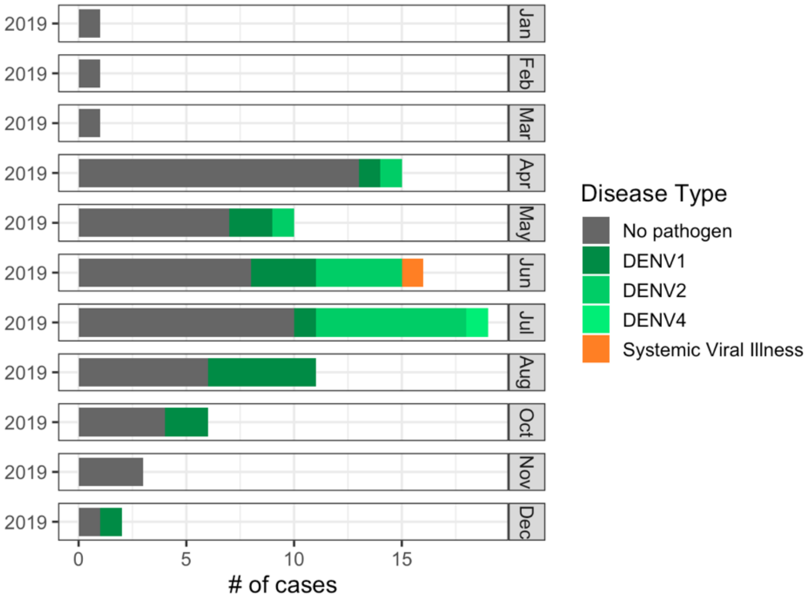

Figure S2. Summary statistics for environmental indices (EIs) by disease outcome type.

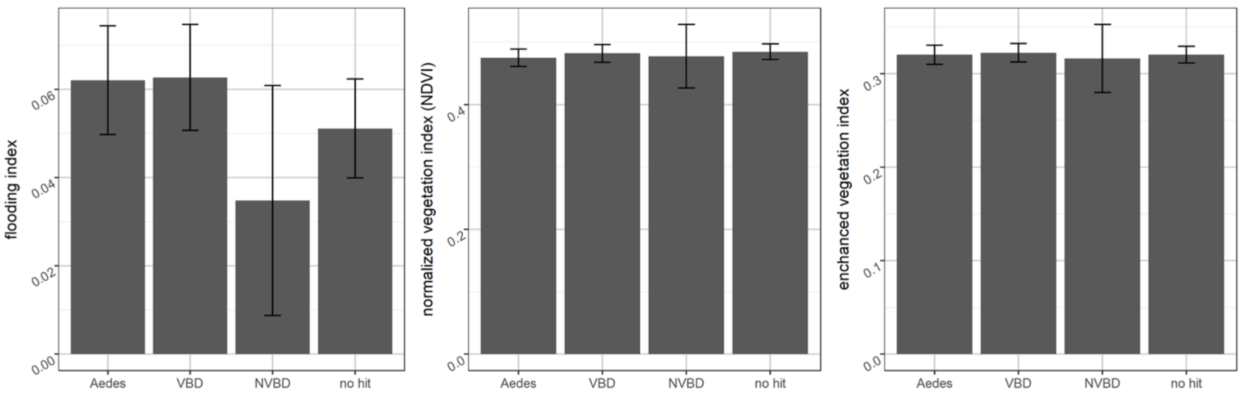

Disease outcome types classified as Aedes = indicates *Aedes spp.*-borne infections (DENV, CHIKV, or ZIKV); VBD = vector-borne diseases (including *Aedes spp.*-borne diseases); NVBD = non-vector-borne diseases; no hit = indicating that no pathogen was identified for these participants). Standard errors were calculated using the t-distribution.

Metagenomics Methods Supplement

The number of raw reads differed greatly between runs on the NovaSeq6000 and iSeq100 (160533 rpM vs 72003 rpM, $p < 0.0001$, respectively). The number of bacterial and parasitic reads were less than viral reads regardless of the sequencing platform (NovaSeq6000 mean rpM of vector-borne disease versus all other clinical categories 171294 vs. 9882, $p < 0.0001$; iSeq100: 93594 vs. 111.7, $p < 0.0001$).

Inclusion of water controls sequenced alongside samples on both the NovaSeq and the iSeq enabled estimation of the number of background reads expected per taxon within the sequencer-specific context. The background correction is discussed under Bioinformatic Analysis (Methods). In particular, due to the sensitivity of the NovaSeq for identifying dengue hits at exceedingly low abundance, a previously developed negative binomial model [described in Mick et al, Nat Comm, Nov 2020] was used to identify cases with NT alignments present at an abundance significantly greater than in the negative water controls and therefore ascertain true pathogen hits from barcode hopping and potential contamination.

Statistical Supplement

Main model

The main model as seen in the main text models the binary response outcome $Y_i \in \{0,1\}$ for $i = 1, \ldots, N$ of whether sera collected from patient i is identified as vector borne disease (VBD) $Y_i = 1$ or not $Y_i = 0$. In logistic regression, we assume the logit of the expected outcome $logit(E[Y_i])$ is a linear combination of the features where $\boldsymbol{\beta}$ is the vector of coefficient values,

$$logit(E[Y_i]) = \mathbf{x}_i\boldsymbol{\beta}.$$

The features used in the model the hospital study for patient i are

$$\mathbf{x}_i^{(Hosp.)} = \left(1, x_{i,flooding}, x_{i,larvicide}, x_{i,age(cat.)}, x_{i,sex}, x_{i,school}, x_{i,car}\right)$$

and the features used in the model for the community study for patient i are

$$\mathbf{x}_i^{(Com.)} = \left(1, x_{i,flooding}, x_{i,larvicide}, x_{i,age(cat.)}, x_{i,sex}, x_{i,school}, x_{i,se}\right),$$

where 1 is used for the intercept, flooding is the smoothed measurement from satellite imagery taken four weeks before the patient's visit, larvicide is a binary response variable indicating whether the household used any larvicide, age is grouped as 0-5 years (the reference group), 5-10 years, 10-18 years, and 18+ years, sex is male or female, attends school is yes or no, car is whether the household owns at least one car, and is the socioeconomic status (self-reported as middle or lower class).

The likelihood of the data $D = \{(\mathbf{x}_i, y_i)\}_{i=1}^{N}$ given the parameters $\boldsymbol{\beta}$ is then

$$L(D|\boldsymbol{\beta}) = \prod_{i=1}^{N} logit^{-1}(\mathbf{x}_i\boldsymbol{\beta})^{y_i} \times (1 - logit^{-1}(\mathbf{x}_i\boldsymbol{\beta}))^{1-y_i}$$

The posterior distribution of $\boldsymbol{\beta}$ is proportional to the likelihood times the prior $\pi(\boldsymbol{\beta})$,

$$P(\boldsymbol{\beta}|D) \propto L(D|\boldsymbol{\beta})\pi(\boldsymbol{\beta}).$$

We assume $\beta \sim MVN(\mathbf{0}, \sigma_i^2\mathbf{I})$ where $\sigma^2 = \sigma_j^2 = 1$ for $i, j \neq 0$ and $\mathbf{I}$ is the identity matrix. We assume the intercept has distribution $\beta_0 \sim N(0, 2.5^2)$. This choice of priors allows for moderately sized tails and is weakly informative. We tried priors of $N(0,1)$ and $N(0, 2.5^2)$, the latter which is the default for stan_glm(). The $N(0,1)$ prior on regression parameters, β_p, in the primary analysis (besides the intercept) has mean 0 and puts 95% of the prior mass for each log odds ratio

between -1.96 and 1.96. The the wider $N(0, 2.5^2)$ prior used in sensitivity analysis has with mean 0 and puts 95% of its prior mass for each log odds ratio between -4.9 and 4.9. This model and all other models mentioned in this text were fit in R using the rstanarm package using the stan_glm() function, which wraps around Rstan and implements the no U-turn variant of Hamiltonian Monte Carlo.[1-3] We used four chains with 10,000 samples per chain.

Adjustment for seasonality

Two features of our data, Scaled Flooding Index and Scaled Vegetation Index were pre-processed before using in the main and following models. For each patient location and using satellite imagery, vegetation and flooding measurements were taken at four buffer points every two weeks over duration of the study. This resulted in 127 point-pairs for each patient. To reduce noise, we smoothed the resulting curves using a thin-plate spline with eight degrees of freedom. Finally, we normalized those points on the splines to produce the smoothed estimates. This transformation reduced noise but preserved seasonality from the two spatial-temporal features. The splines were fit using the gam package in R.[4]

Featured selection

To select which features would be in the model, we used expert knowledge, exploratory plots and tables, and simulation. Feature selection was performed for the community study since it had a larger data set. The features in this model were then also used (when possible) in the hospital data set. Features we considered were, scaled flooding index (4-week lagged), scaled vegetation index (4-weeks lagged), age (0-5, 5-10, 10-18, and 18+ years), sex, whether the individual attends school, socio-economic status (self-reported), whether the family owns a car, insecticide use, larvicide use, mosquito net use, coils use, animal contact, insect contact, and post March 15, 2021 (a proxy for the COVID-19 pandemic start date). Symptom data such as temperature, rash appearance, runny nose, or cough were not included for features in our model.

Model Diagnostics

In Statistical Supplemental Table 1, we show the results of our main model with two choices of priors for the coefficients: $N(0,1)$ and $N(0, 2.5^2)$. The former choice induced narrower posterior

credible intervals for estimates of log odds ratios. The alternative prior choice does not change which 95% credible intervals for the estimated odds ratio include 1.

Statistical Supplemental Table 1. Sensitivity to priors of posterior distributions of the OR of the features. We see that the model with the N (0,1) prior has a smaller range of OR than the N (0, 2.25^2) prior.

Feature	Hospital OR		Community OR	
	N(0,1)	N(0,2.25^2)	N(0,1)	N(0, 2.25^2)
Intercept	0.14 (0.06, 0.31)	0.12 (0.05, 0.29)	0.38 (0.04, 3.2)	0.32 (0.02, 3.64)
Scaled Flooding Index (4-week lag)	1.22 (0.94, 1.61)	1.22 (0.93, 1.62)	2.03 (1.24, 3.51)	2.15 (1.27, 3.89)
Uses Larvicide	0.32 (0.11, 0.8)	0.22 (0.06, 0.67)	0.99 (0.38, 2.6)	1.02 (0.33, 3.22)
Age 5-10 years	2.35 (1.11, 5.06)	2.91 (1.24, 6.86)	1.19 (0.37, 3.92)	1.22 (0.24, 6.21)
Age 10-18 years	2.68 (1.4, 5.29)	3.25 (1.54, 6.86)		
Age 18+ years	1.18 (0.6, 2.32)	1.32 (0.64, 2.73)		
Female	1.16 (0.73, 1.83)	1.16 (0.73, 1.86)	1.11 (0.46, 2.75)	1.16 (0.43, 3.31)
Attends School	1.34 (0.74, 2.44)	1.26 (0.66, 2.43)	1.26 (0.4, 4.04)	1.32 (0.27, 6.62)
Household Has a Car	1.95 (1.19, 3.21)	2.01 (1.21, 3.37)		

Feature	Hospital OR		Community OR	
	N(0,1)	N(0,2.25^2)	N(0,1)	N(0, 2.25^2)
Middle Class (vs. Lower)			1.22 (0.41, 3.73)	1.28 (0.35, 5.02)

We show the pairs plot (excluding the age bins for visibility of the figure) for the Hospital model (Fig. 1). Generally, the posterior pairs plots seem to have no correlation with perhaps the exception of having a car and the scaled flooding index, which may have a small, negative linear trend.

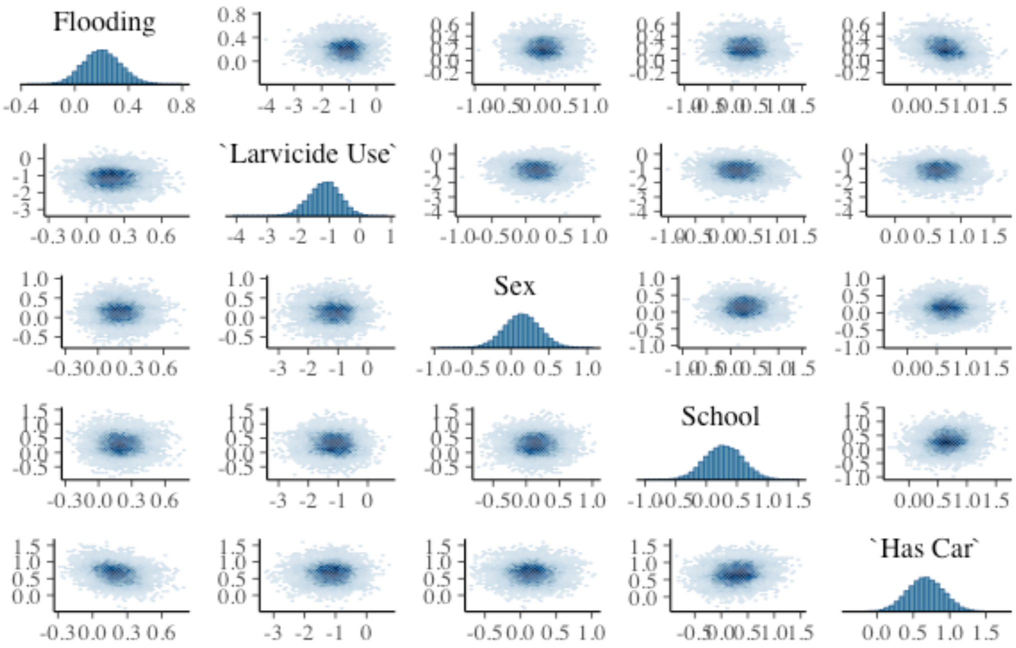

Statistical Supplemental Figure 1. Posterior pairs plot for the hospital model. For all pairs except perhaps car and flooding we do not see any linear correlation.

We show the posterior predictive plot (Fig. 2) for both the community and hospital data sets. These plots show how well our model can separate high risk of VBD individuals from lower risk ones. We first group the individuals into five quintiles based on their posterior median probability of VBD from the model. Then for each of the 10,000 posterior predictions we find the average posterior prediction for the given quintile. We then plot the 95% CI for each quintile over the 10,000 quintile average predictions. First, we note that each of the observed proportion VBD (the

horizontal dashed lines) intersects with the quintile range of the same color, indicating that our model predictions by quintile category match the observed values well. The second thing we notice is that the proportion VBD by quintile increases with the quintile, suggesting that our model does a good job of stratifying who is at higher risk.

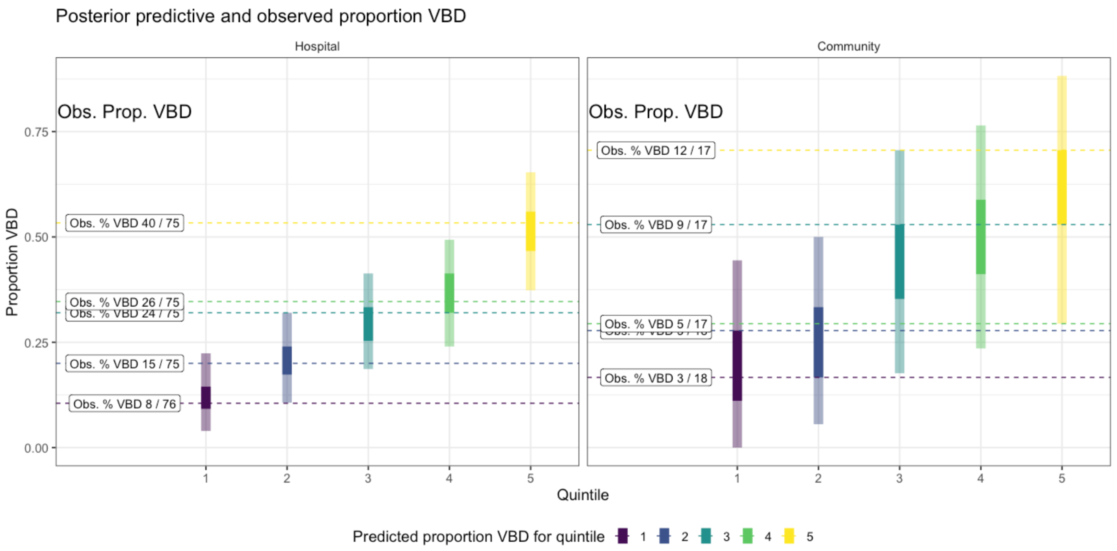

Statistical Supplement Figure 2. Posterior predictive plot for the Hospital and Community studies. The five colors denote quintiles that are formed from the posterior median predictions for each individual in the study. The vertical bars show the 95% CI (and 50% CI as a darker line) of the average posterior prediction for each individual in the quintile. The dashed horizontal lines show the observed values of VBD in each quintile. Since the vertical lines intersect with the horizontal lines of the same quintile, we see that our model is a good fit.

Finally, we show the computational statistics for modeling fitting (Statistical Supplemental Table 2). In our sampler, we used 4 chains with 10,000 samples each. The effective sample size is over 10,000 for each parameter and over 8,000 for the log posterior and all potential scale reduction factors are approximately 1. Hence, we conclude that our sampler has converged and drawn an adequate sample from the target posterior.

Statistical Supplemental Table 2. Effective sample size and convergence for our main model. Four chains were run each with 10,000 samples.

Feature	Effective Sample Size	Rhat
(Intercept)	20,297	1.0001
NFI_lag_4wks_scale	20,122	0.9999
rf_larvicide	18,795	0.9998
age_bin2	13,296	1.0001
age_bin3	13,141	1.0001
age_bin4	17,104	1.0000
dem_gender	20,882	1.0000
dem_school	14,807	1.0000
dem_car	19,827	1.0001
mean_PPD	20,856	1.0001
log-posterior	8,315	1.0001

Other models

We also tried other models with different sets of features and show two such examples here. In addition to the main model, we show the coefficient median and 95% CIs in a more parsimonious model and a more complex model. These results are displayed in Table 3 and 4. In the more parsimonious model, we use a slightly smaller feature set, using only the variables that did not contain 1 in the 95% CI of the posterior OR in addition to demographic variables of age bin and sex. These non-demographic features for the hospital data are larvicide use and having a car. For the community data, the only non-demographic feature not including 1 in the 95% CI of the posterior is scaled flooding index. The results of these more parsimonious models result in coefficient posterior medians are very similar to one another.

In the more complex model, we use all the features in the main model in addition to the interaction of age bin and sex. Again, the coefficient estimates are quite similar to the main model. Additionally, these more complex models do not seem to result in more interpretable models nor contradict the results of the main models.

Statistical Supplemental Table 3. Hospital study. Median coefficients and 95% CI for the main, more parsimonious, and more complex model.

Feature	More parsimonious	Main	More complex
Intercept	0.14 (0.06, 0.31)	0.14 (0.06, 0.31)	0.14 (0.06, 0.32)
Uses Larvicide	0.33 (0.12, 0.83)	0.32 (0.11, 0.8)	0.32 (0.11, 0.83)
Age 5-10 years	2.75 (1.43, 5.41)	2.35 (1.11, 5.06)	2.68 (0.96, 7.37)
Age 10-18 years	3.22 (1.83, 5.69)	2.68 (1.4, 5.29)	2.64 (1.21, 5.86)
Age 18+ years	1.18 (0.61, 2.27)	1.18 (0.6, 2.32)	1.18 (0.6, 2.32)
Female	1.16 (0.74, 1.82)	1.16 (0.73, 1.83)	1.15 (0.72, 1.84)
Household Has a Car	2.1 (1.32, 3.34)	1.95 (1.19, 3.21)	1.95 (1.19, 3.23)
Scaled Flooding Index (4-week lag)		1.22 (0.94, 1.61)	1.22 (0.94, 1.62)
Attends School		1.34 (0.74, 2.44)	1.43 (0.53, 3.65)
Age 5-10 years and School			0.8 (0.23, 2.78)
Age 10-18 years and School			0.96 (0.32, 3)
Age 18+ years and School			1.06 (0.2, 5.19)

Statistical Supplemental Table 4. Community study. Median coefficients and 95% CI for the main, more parsimonious, and more complex model.

Feature	More parsimonious	Main	More complex
Intercept	0.46 (0.18, 1.13)	0.38 (0.04, 3.2)	0.38 (0.04, 3.17)
Uses Larvicide		0.99 (0.38, 2.6)	0.99 (0.38, 2.66)
Age 5-10 years	1.39 (0.56, 3.49)	1.19 (0.37, 3.92)	1.19 (0.32, 4.24)
Female	1.12 (0.46, 2.7)	1.11 (0.46, 2.75)	1.25 (0.31, 5.1)
Scaled Flooding Index (4-week lag)	2.02 (1.27, 3.4)	2.03 (1.24, 3.51)	2.04 (1.23, 3.53)
Attends School		1.26 (0.4, 4.04)	1.12 (0.46, 2.76)
Middle Class (vs. Lower)		1.22 (0.41, 3.73)	1.21 (0.41, 3.72)
Age 5-10 years and School			1.03 (0.22, 4.59)

In summary, we attempted to run both more complex and less complex models, using different sets of features available. We found that our main models seem to have consistent coefficient estimates with respect to both sets.

Statistical Supplement References

1. Brilleman, SL, MJ Crowther, M Moreno-Betancur, J Buros Novik, and R Wolfe. 2018. “Joint Longitudinal and Time-to-Event Models via Stan.” https://github.com/stan-dev/stancon_talks/

2. R Core Team. 2020. R: A Language and Environment for Statistical Computing. Vienna, Austria: R Foundation for Statistical Computing. https://www.R-project.org/.

3. Stan Development Team. 2020. “RStan: The R Interface to Stan. https://mc-stan.org/

4. Hastie, Trevor. 2020. Gam: Generalized Additive Models. https://CRAN.R-project.org/package=gam.
